# Advances in non-coding RNA in tendon injuries

**DOI:** 10.3389/fgene.2024.1396195

**Published:** 2024-05-21

**Authors:** Bin Wang, Qiang Chen, Xiaodi Zou, Ping Zheng, Jie Zhu

**Affiliations:** ^1^ Department of Plastics, Tiantai People’s Hospital of Zhejiang Province (Tiantai Branch of Zhejiang Provincial People’s Hospital), Hangzhou Medical College, Taizhou, China; ^2^ Center for Plastic and Reconstructive Surgery, Department of Hand and Reconstructive Surgery, Zhejiang Provincial People’s Hospital, Affiliated People’s Hospital, Hangzhou Medical College, Hangzhou, China; ^3^ Department of Orthopedics, The Second Affiliated Hospital of Zhejiang Chinese Medical University, Hangzhou, China; ^4^ Center for General Practice Medicine, Zhejiang Provincial People’s Hospital, Affiliated People’s Hospital, Hangzhou Medical College, Hangzhou, China

**Keywords:** tendon injuries, healing capacity, treatment modalities, non-coding RNAs, functional recovery

## Abstract

Tendons serve as important weight-bearing structures that smoothly transfer forces from muscles to skeletal parts, allowing contracted muscle movements to be translated into corresponding joint movements. For body mechanics, tendon tissue plays an important role. If the tendons are damaged to varying degrees, it can lead to disability or pain in patients. That is to say, tendon injuries havea significant impact on quality of life and deserve our high attention. Compared to other musculoskeletal tissues, tendons are hypovascular and hypo-cellular, and therefore have a greater ability to heal, this will lead to a longer recovery period after injury or even disability, which will significantly affect the quality of life. There are many causes of tendon injury, including trauma, genetic factors, inflammation, aging, and long-term overuse, and the study of related mechanisms is of great significance. Currently, tendon there are different treatment modalities, like injection therapy and surgical interventions. However, they have a high failure rate due to different reasons, among which the formation of adhesions severely weakens the tissue strength, affecting the functional recovery and the patient’s quality of life. A large amount of data has shown that non coding RNAs can play a huge role in this field, thus attracting widespread attention from researchers from various countries. This review summarizes the relevant research progress on non-coding RNAs in tendon injuries, providing new ideas for a deeper understanding of tendon injuries and exploring new diagnostic and therapeutic approaches.

## Introduction

During exercise and work, a large number of tendon injuries occur, which can cause pain interference for patients and even lead to disability. Besides tendon injuries, they also occur on achilles tendons or flexor tendons. There are 300,000 newly diagnosed cases of tendon injuries worldwide each year (Clayton and Court-Brown, 2008). Research has shown that tendon injuries can be classified into two types: chronic and acute, which can be caused by both external and internal factors in the body. Tendon injuries can be combined or isolated. Acute trauma and chronic diseases are respectively influenced by external and internal factors (Sharma and Maffulli, 2006a). The relevant indicators mainly include changes in cross-sectional area, inflammation, and pain. In addition, there are histological changes such as enhanced blood vessels, increased cell count, or increased proteoglycans. Recent research data shows that most acute tendon injuries exhibit histological changes similar to chronic tendon disease, and the underlying mechanisms are worthy of further investigation (Voleti et al., 2012). Because the metabolic rate of this area is relatively low, the healing time is relatively long, usually taking more than a year (Lu et al., 2017). Although there are many studies on functional prognosis, the current treatment methods used are still relatively limited, mainly including surgical treatment and conservative treatment, which greatly affect the quality of life of patients (Andres and Murrell, 2008). Therefore, there is a need to find new and more effective treatments to cure tendon injuries.

**FIGURE 1:**
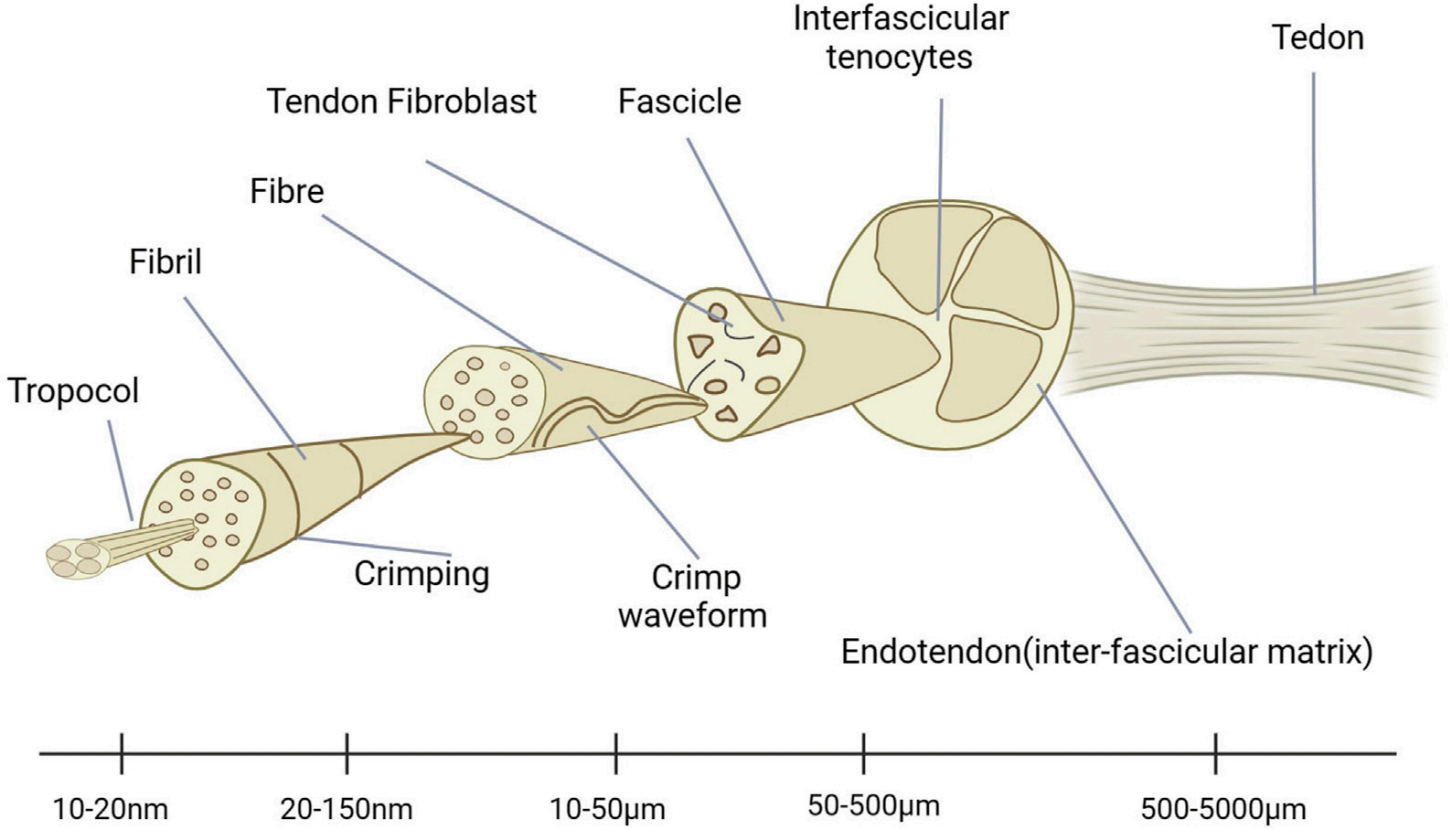
Ultrastructure of tendons.

There are very complex structures in tendons. It plays a very important role in the entire human body. Through the movement of tendons, strength can be effectively transmitted, ultimately achieving the various movements required by the human body. Therefore, the study of its kinematics and pathology is very important. By analyzing its hardness and other mechanical properties, it is known that it can buffer the phenomenon of stress concentration. By releasing and storing energy, it can improve the exercise efficiency. However, due to their viscoelasticity, tendons also expend energy and may help protect bones and muscles from injury (Voleti et al., 2012). We can grasp the compositional characteristics of tendons through ultrastructural analysis (Figure 1) and discover many constituent units within them. These data have been extensively reported in early literature. Through research, it is known that there are shallow aponeuroses near the tendon unit that can effectively reduce friction. This hierarchical structure allows all structures to be horizontally parallel to the long axis of the tendon, making it ideally suited to carry and transmit large tensile mechanical loads (Wang et al., 2012).

Tendons contain various components that have different functions. It should be noted that these substances have different functions. Among them, the content of collagen is very high, and the highest is type I collagen, which accounts for about 70% (Jh, 2006). I Related research structures show that tendons also contain a certain proportion of glycoproteins and proteoglycans (Winnicki et al., 2020). The main reason why tendons have good viscoelasticity is due to the combined action of the aforementioned components (Elliott et al., 2003). In addition, there are also various cells present in tendons, including tendon fibroblasts, synoviocytes, and vascular cells (Wang et al., 2012). The latter is also the most important type of cell, which is usually distributed in rows between collagen fiber bundles. Its function is to synthesize various extracellular matrix proteins, thereby forming the collagen matrix, which can achieve tendon remodeling during healing, making its role crucial (Jh, 2006).

At present, there are still some unclear issues regarding the specific causes of tendon injuries. The current main viewpoint is that it is the result of internal and external factors. If Sohu is affected by various external factors (such as excessive use of tendons during sports training), it may lead to serious tendon damage, weakness, and pain in certain parts of the body. Studies have shown that intrinsic factors also include tooth degeneration (Jamil et al., 2017). From a clinical perspective, as a person’s age gradually increases, the probability of tendon injury also gradually increases (Tashjian, 2012). Through a series of animal experiments, it was also found that this factor is closely related to the activity of matrix metalloproteinases (MMPs) and changes in the number of live tendon cells (Yu et al., 2013). Meanwhile, gender is also an inherent influencing factor for tendon degeneration, with female tendons exhibiting decreased collagen synthesis rates during acute exercise and inhibited hypertrophy during habitual exercise (Magnusson et al., 2007). In addition, systemic diseases, genetic variants, fluoroquinolone antibiotic use, smoking, hypercholesterolemia, and family history predispose to tendon injury (Tashjian, 2012; Magnan et al., 2014).

## Tendon healing

The research results on tendon healing channels indicate that they are related to both intrinsic mechanisms and extrinsic factors (Loiselle et al., 2016; Titan et al., 2019). As tendon cells undergo division and gradually move to severely damaged areas, internal healing will gradually be achieved. There is research confirming that when synovial tissue and peripheral sheath cells are invaded, exogenous healing occurs (Voleti et al., 2012). There are a series of factors that affect the balance between these two methods. Among them, endogenous healing has good mechanical properties and fewer complications. In addition, exogenous healing is mainly related to factors such as scars. In the process of tendon healing, the first step is to have a brief interaction with inflammatory cells and tendon cells invading the original wound. When the human body is injured, cell death occurs, leading to initial inflammation. Inflammatory cells, such as neutrophils and macrophages, will slowly infiltrate the damaged area. Then, fibroblasts gradually proliferate and enter the damaged area of the body, while collagen fibers gradually deposit after completing proliferation. The remodeling phase of tendon repair is a crucial period during which the initial, structurally weaker type III collagen is substituted with the more robust type I collagen. This procedure is crucial for the restoration of the tendon’s structural integrity and mechanical strength. During the remodeling phase, which can range from 6 weeks to 12 months, there is a conversion from type III to type I collagen. During the proliferation stage of healing, Type III collagen is formed, exhibiting high water content and concentrations of glycosaminoglycans (Ischenko et al., 2017). During the remodeling process, there is a phase of consolidation in which the tenocytes and collagen fibers align themselves in the direction of stress. Additionally, a larger amount of type I collagen is produced. The transition is critical because type I collagen possesses superior tensile strength and is the prevailing collagen type in undamaged tendons. The alignment of cells, specifically tenocytes, plays a crucial role in determining the deposition of collagen along the lengthwise direction of the tendon. Tenocytes are responsible for producing the extracellular matrix (ECM) and collagen fibers. Ensuring that tenocytes align correctly with the axis of mechanical stress is crucial for the development of structured collagen fibers that have the ability to sustain tensile stresses (Gigante et al., 1996). The alignment of tenocytes, specifically responsible for producing the extracellular matrix (ECM) and collagen fibers, affects the deposition of collagen along the lengthwise direction of the tendon. Ensuring that tenocytes are correctly aligned along the axis of mechanical stress is crucial for the development of structured collagen fibers that are capable of withstanding tensile stresses (Sharma and Maffulli, 2006b). Through analysis, it can be found that there is a significant cross-linking phenomenon in collagen fibers, which enhances the strength of regenerated tissue. These phenomena also occur during the healing process of tendons. Some teams have studied the biological changes that occur in fibroblasts during these processes. At this point, collagen will become more mature fibers and undergo reorganization. Tendon fibroblasts can jointly control the above processes through MMPs, growth factors, and cytokines (James et al., 2008).

## Complications of tendon healing

### Scarring

The process of reshaping can be divided into different stages, gradually becoming mature after development (James et al., 2008). In the first stage, the cellular tissue undergoes complex transformation, ultimately becoming fibrous tissue. After entering the mature stage, fibrous tissue will undergo transformation and form scar tissue, during which vascular function and tendon cell metabolism will decrease to varying degrees (Sharma and Maffulli, 2005). The scar tissue formed by this process has significant differences in biomechanical properties compared to natural tendons: it has lower strength and higher hardness, and is prone to adhesion. After healing, the performance of tendon tissue has decreased to varying degrees compared to before (Bruns et al., 2000).

### Adhesion

When exogenous healing occurs, scar tissue may adhere to each other, thereby affecting the sliding process of tendons. The research results show that the main reason is surgical repair and initial injury leading to adhesion formation (Jaibaji, 2000). Myofibroblasts are also an important factor in the process of tendon adhesion, and their biological mechanisms are particularly interesting (Weiler et al., 2002). The scope and degree of adhesion mainly include four levels: level 0 means no adhesion occurs at all; Grade I represents a small thickness of adhesion and no blood vessels; Grade II corresponds to a thicker finger adhesion, no blood vessels, and only appearing at the anastomotic site; Level III corresponds to thicker adhesions, presence of blood vessels, and a relatively large range (Voleti et al., 2012). Because they can lead to the loss of sliding mechanisms, they will result in functional impairments.

## Strategies to promote tendon healing

### Physical stimulation

Biophysical stimulation refers to a range of therapeutic modalities to assist tendon healing, but is limited to well-vascularized tissues that have a high likelihood of healing without surgery, or as an adjunct to surgery (Benazzo et al., 2008). The above therapies mainly include ultrasound therapy (Lu et al., 2016), magnetic field therapy (Xu et al., 2014; Prucha et al., 2018), cryotherapy (G et al., 2013), and physical therapy (G et al., 2013), each with its own advantages and characteristics.

### Growth factors

In recent years, scholars have pointed out that many growth factors have a significant impact on the growth and changes of tendons. These factors mainly include bFGF, (GDF) −5, GDF6, GDF7, IGF1, PDGF, (TGF)- β 1 and VEGF et al. (Leong et al., 2020). Exogenous growth factors act to increase the speed of the entire process from ECM synthesis to cell proliferation. Therefore, it helps to quickly complete the repair process (Y et al., 2023). At the same time, extracellular regulated protein kinase (ERK) 2 also participates in regulating (FGF-2) and TGF- β Induced fibroblast proliferation and collagen expression (Ruan et al., 2013). There are also research results showing that vascular endothelial growth factors IGF1 and PDGF can also affect processes such as cell proliferation, adhesion, angiogenesis, and matrix formation, which are beneficial for the smooth healing of tendons (Ilaltdinov et al., 2021). Growth factors can be released through a series of methods, including overexpression vectors, and research on these methods has high academic significance. These growth factor treatments appear to have some benefit for tendon healing, especially in the early stages of tendon and ligament healing, but the long-term benefits appear to be limited (Liu et al., 2011). The complexity of growth factor treatment strategies significantly increases due to factors such as residence time, administration time, dosage, and synergistic effects (Thomopoulos et al., 2015).

### Epitenon tenocytes

Physical preparations mainly serve as physical barriers for tendons and other tissues, which can reduce the degree of adhesion. Related drugs mainly include 5-fluorouracil, hyaluronic acid, and corticosteroids (Voleti et al., 2012). Recent studies have shown that the use of bioactive adjuvants for the treatment of various sports injuries has enormous practical value. Especially the application of platelet rich plasma (PRP) has received much attention. As an autologous blood product, it can effectively remove red blood cells by extracting peripheral venous blood from the human body and then centrifuging it, leaving only plasma and platelets (Southworth et al., 2019). PRP contains almost all of the growth factors necessary for tissue healing and is currently used in a variety of tendon and ligament injuries such as rotator cuff tendinosis disease and partial tear of the ulnar collateral ligament (Leong et al., 2020). A research group conducted a randomized controlled trial using the highly innovative PRP enhanced patellar tendon autotransplantation for anterior cruciate ligament reconstruction. Through comprehensive analysis and comparison, it was found that the results were not significant (Kia et al., 2018). Although there is currently relatively little high-level evidence, it should be recognized that PRP is a very important conservative therapy (Leong et al., 2020).

### Organizational engineering

Tissue engineering can be used to promote tendon healing, and related research is worth conducting in depth. The research on the potential mechanisms of mesenchymal stem cells has gradually increased in recent years, and many teams have explored this topic. The current common view is that it can be applied to the transportation process of damaged areas to achieve good repair results. This is of great significance for clinical applications. A recent paper has reported the issue of using tissue engineered tendons to effectively bridge tendon defect areas (Young et al., 1998). Implantation of decellularized tendons into tendon sheath fibroblasts or epitenon tenocytes will facilitate tendon regeneration, and the mechanism behind it has been analyzed (Kryger et al., 2007). Biomodification of decellularized allogeneic flexor tendons reduces adhesions and increases postoperative joint mobility, making them as good as or better than extra-synovial autografts (Drake et al., 2013). In animal models, the biomechanical properties of decellularized tendon grafts can be enhanced by applying periodic mechanical stimulation (Klifto et al., 2018). In addition, the nanofibers prepared by electrospinning have controllable diameter, orderly arrangement, high porosity, and a large specific surface area, which are usually easy to meet the requirements of tissue engineering for tendon repair, with a low production cost and high efficiency, which makes them ideal for tendon injury treatment (Li et al., 2022; Weng et al., 2022). By optimizing the design and fabrication methods, electro spun 3D scaffolds can function in the three phases of inflammation, proliferation, and remodeling after tendon injury (Y et al., 2023). Hydrogels can provide various beneficial matrices for tendon healing, and can also accurately deliver biological agents, so they have important application potential in the field of tendon injury treatment (Hu et al., 2023). In fact, the biggest advantage of tissue engineering methods is to provide mechanical support for the initial stage of tendon injury. To further promote tendon and ligament healing, careful consideration needs to be given to the selection of cells and the type of biomaterial scaffolds that can greatly influence the outcome of the treatment (Leong et al., 2020).

## Non-coding RNAs are involved in regulating the development of tendon injuries

Non-coding RNAs (ncRNAs) are a group of RNA molecules that lack the ability to produce proteins. They participate in diverse biological processes, such as the regeneration of tendons. Non-coding RNAs can be classified into two primary categories: microRNAs (miRNAs) and long non-coding RNAs (lncRNAs). They have a significant impact on tendon repair as they control inflammation, stimulate cell growth and specialization, and affect the restructuring of the extracellular matrix (Nemeth et al., 2024). miRNAs are short RNA molecules that control gene expression by attaching to messenger RNA (mRNA) molecules. They have a vital function in the healing of tendons by regulating inflammation and stimulating the growth and specialization of cells. One specific example is miR-15b-5p, a type of microRNA that is located after circRNA-Ep400. It is found at higher levels in M2 macrophage exosomes and has a role in promoting peritendinous fibrosis following tendon injury. This is achieved by modulating the FGF-1/7/9 pathway (Zhang et al., 2023). Long non-coding RNAs (lncRNAs) are RNA molecules that exceed 200 nucleotides in length and do not possess the ability to produce proteins. They have the ability to function as competitive endogenous RNAs (ceRNAs) by forming a bond with miRNAs, thereby controlling their activity. One specific example is circRNA-Ep400, which is a type of circular RNA that is found in higher levels in M2 macrophage exosomes. This circRNA plays a role in promoting peritendinous fibrosis following tendon injury by modulating the FGF-1/7/9 pathway (Zhang et al., 2023).

## MiRNA and tendon injury

### Overview of miRNA

**FIGURE 2:**
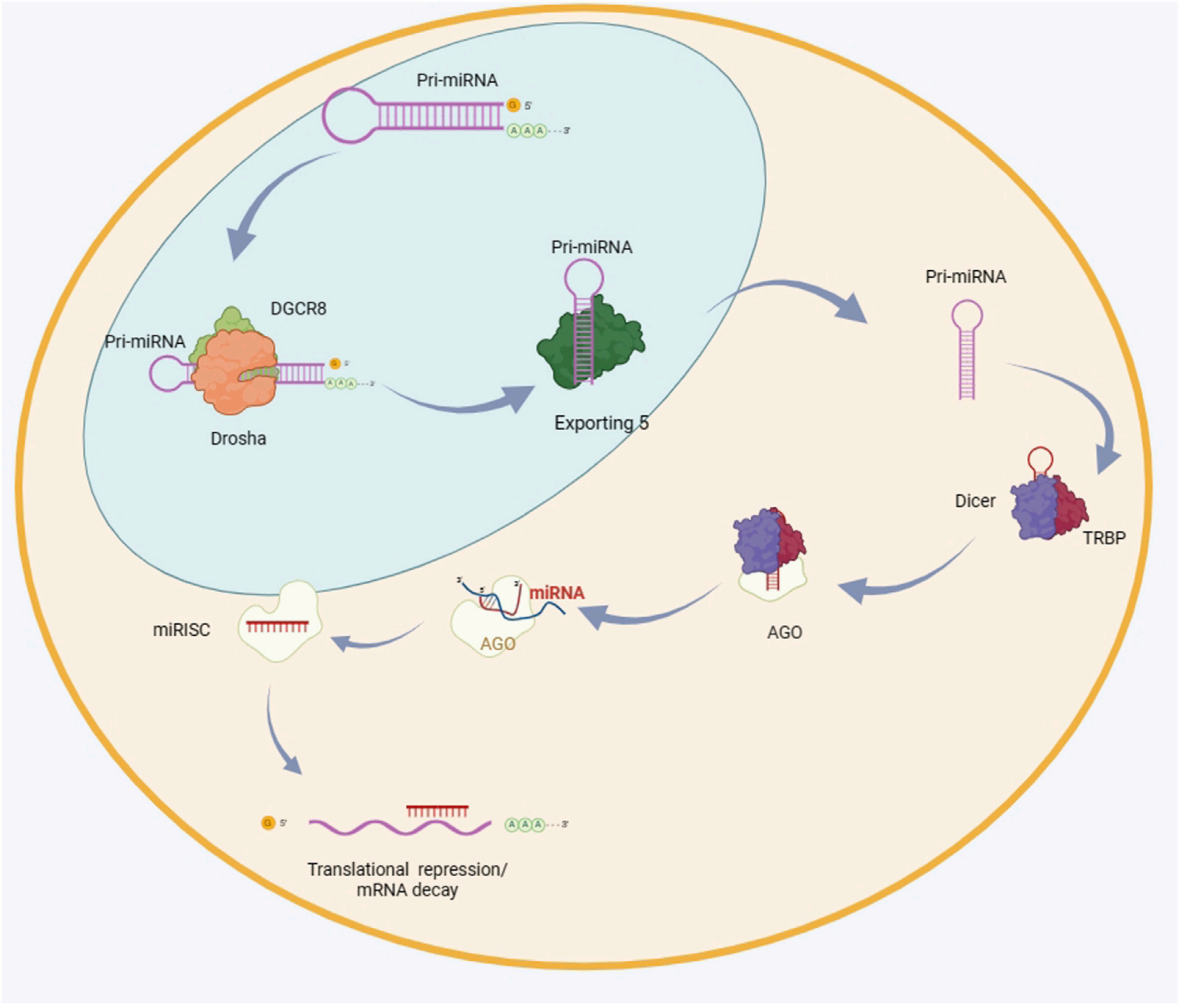
The biogenesis of miRNA. The primary microRNA (pri-miRNA) undergoes the first cleavage by the enzymes Drosha and DGCR8, resulting in the formation of a precursor microRNA (pre-miRNA) that possesses a hairpin structure. The pre-miRNA molecule is transported to the cytoplasm by Exportin 5 and undergoes further processing by Dicer and TRBP. The mature miRNA duplex undergoes unwinding and enters the RNA-induced silencing complex (RISC) by interaction with AGO proteins. The RISC (RNA-induced silencing complex) interacts with the 3′untranslated region (UTR) of the target mRNA, leading to either inhibition of translation or destruction of the mRNA. DGCR8 is an acronym for DiGeorge syndrome critical region 8. TRBP stands for transactivation response element RNA-binding protein. RISC refers to RNA-induced silencing complex. AGO is short for argonaute. UTR stands for untranslated region.

Many researches show that normally miRNA is highly expressed in different species (Bartel, 2004). There are currently around 2600 mature miRNAs discovered. They play an important role in the regulation of many coding genes, and relevant data can be found in recent reports (Friedman et al., 2009). Partial complementarity can be achieved by binding to the target mRNA. In addition, miRNA can also effectively control a large number of target genes. It can be inferred that the changes in miRNA expression will significantly affect the entire process of cell proliferation to apoptosis. The regulatory role of miRNAs in tissue regeneration has been revealed through the observation of fetal skin healing patterns in mice at different developmental stages (Cheng et al., 2010). A large amount of research data indicates that the issue of miRNA expression imbalance is very important, and it is closely related to various diseases, and its main mechanisms have been explored (Hébert and De Strooper, 2007; Dong et al., 2009; Heller et al., 2017). It plays an important role in maintaining organizational stability, and in recent years, multiple teams have been involved in exploring this field and have achieved some key breakthroughs (Hébert and De Strooper, 2007; Thankam et al., 2018; Moro et al., 2019). The biogenesis of miRNA is demonstrated in Figure 2 (Liu et al., 2021).

### Role of miRNAs in tendon injury

Several studies have demonstrated the important role of miRNAs in mediating tendonogenesis, tendon cell apoptosis, or senescence (Poulsen et al., 2014; Chen et al., 2015; Liu et al., 2019). For example, Wang et al. found that miR-124-targeted inhibition of early growth responsive gene-1 (EGR1) resulted in reduced collagen formation in human tendon-derived stem cells during tendonogenesis (Wang et al., 2016); Chen et al. showed that miR-135a can serve as an indicator of tendon cell death by increasing the expression of tendon marker genes Scx (scleraxis) and Tnmd (tenomodulin). These genes are important for cell specialization and contribute to the development of tendon-like cells in rat tendon-derived stem cells. (Chen et al., 2015); in another study using mouse tendon-derived stem cells, overexpression of miR-378a led to a significant reduction in collagen synthesis and tendonogenic gene expression, which was further confirmed by targeting of TGF-β2 further confirmed the regulatory inhibition of tendonogenesis by miR-378a (Liu et al., 2019). Ageing is thought to have a negative impact on tendon composition and function. A comprehensive RNA expression analysis of young and aged human Achilles tendons found that miR-1245a expression was suppressed in aged Achilles tendons compared to young Achilles tendons (Peffers et al., 2015). It was shown that human umbilical cord mesenchymal stem cell-derived exosomes (HUMSC-Exos) containing low levels of miR-21a-3p reduced the proliferation of rat fibroblasts *in vitro* and inhibited fibrogenic genes type III collagen and α-smooth muscle-actin (α-SMA) expression; and in a rat tendon adhesion model, topical application of HUMSC-Exos helped alleviate tendon adhesions (Liu et al., 2021). Studies have demonstrated the potential therapeutic role of miR-29a delivery in promoting tendon remodeling and improving tendon healing after injury (Liu et al., 2021). As a hypovascular tissue, angiogenesis is a key factor in tendon healing. Studies have shown that some miRNAs can effectively regulate the process of angiogenesis, and further analysis has found that miR-210 mediated angiogenesis is mainly limited to early tendon healing (Usman et al., 2015). In addition, Xu et al., used rats as animal models to deeply explore the role of VEGFA in tendon bone healing, and provided constructive suggestions for the mechanism involved (Xu et al., 2019). miR-144-3p plays a significant role in regulating tenocyte proliferation and migration during tendon repair (Song et al., 2022). It is enriched in exosomes derived from tendon stem cell (TDSCs)and promote these cellular processes by targeting AT-rich interactive domain 1A (ARID1A) (Lyu et al., 2023). On the other hand, it upregulates PTEN, which is involved in the PTEN/mTOR/TGF-β1 signaling cascades, crucial for tendon healing (Song et al., 2022). Additionally, miR-114-3p acts as a key regulator in the repair, aiding in tendon-specific marker expression and protection against oxidative stress (Song et al., 2022). Therefore, it, through its regulatory effects on tenocytes, emerges as a promising target for promoting tendon repair and regeneration.

MiRNA based therapies mainly include two types: miRNA replacement therapy and miRNA inhibition therapy, each with its own advantages. Among them, the latter is mainly achieved through miRNA inhibitors. MiRNA replacement therapy is carried out by introducing miRNA mimics into the cell to act as an exogenous source of additional miRNAs (Rupaimoole and Slack, 2017). The success of miRNA therapy mainly lies in its effective localization and controlled delivery to the desired tissue or organ (Mencía Castaño et al., 2015). Scaffold-based systems have the advantage of achieving site-specific miRNA delivery, which can avoid the off-target effect of miRNA therapy and thus maximize the function of miRNAs. In addition, natural degradation of the scaffold ensures sustained release of the encapsulated or immobilized miRNA. Recently, various biomaterials have been developed as novel delivery platforms for miRNA therapy, such as hydrogels, collagen-nanohydroxyapatite composites, electrospun scaffolds, and microspheres (Conde et al., 2016; Zhang et al., 2016; Zhou et al., 2016). More importantly, a variety of bioscaffolds with multiphase structures that highly recapitulate the tendon ECM microenvironment or gradient tendon-bone interface have been developed and shown favorable results (Liu et al., 2017; Ker et al., 2018; Zhu et al., 2018; Liu et al., 2019; Wang et al., 2021). Besides, there are various delivery systems for non-coding RNA. Exosomes, which are a specific kind of vesicle found outside of cells, have become a highly promising method for delivering medicines, including non-coding RNA. Naturally occurring vesicles have the ability to encapsulate miRNAs and lncRNAs, providing protection against degradation and aiding in their distribution to specific cells (Zou et al., 2023). Novel delivery systems utilising nanoparticles have been created to enhance the dispersion and effectiveness of non-coding RNA treatments. An example is the development of a medication delivery system that uses nanoparticles to specifically target tendons and pharmacologically regulate tendon repair. This technique utilises spatial transcriptomics data to pinpoint precise targets within the repairing tendon, such as Acp5 (Adjei-Sowah et al., 2023). Therefore, we can be optimistic that the combination of biomimetic scaffolds and miRNA delivery can play a beneficial role in regulating tissue repair and regeneration after tendon injury.

## Long-stranded non-coding RNA and tendon injury

### Overview of long chain non-coding RNAs

Long non coding RNA (lncRNA) has an open reading frame and plays a regulatory role in certain cellular biological processes, which has been confirmed by experimental results (Chen and Zhao, 2014; Quinn and Chang, 2016). lncRNAuc. 134 inhibits the progression of hepatocellular carcinoma by suppressing Culin 4A (CUL4A)-mediated ubiquitination of Large Tumor Suppressor Kinase 1 (LATS1) (Ni et al., 2017). lncRNA KCNQ1OT1 is overexpressed in mesenchymal stem cells, and lncRNA KCNQ1OT1 is protective against myocardial ischemia/reperfusion injury (Golding et al., 2011; Li et al., 2017). Some research teams have shown that LncRNA plays a regulatory role in tissue regeneration, and related mechanisms have also been widely explored. In addition, some evidence also suggests that it can affect the differentiation process of cells (Fatica and Bozzoni, 2014).

### Role of long chain non-coding RNA in tendon injury

In recent years, lncRNA research has progressed rapidly. Previous studies have reported that many human diseases, such as orthopedic and cancer diseases, are associated with lncRNA deficiency, mutation, or overexpression. Studies have highlighted several lncRNAs with significant potential in tendon healing. Amony these, XIST, H19, MALAT1, KCNQ1OT1, and AC108925 have shown promise in promoting tendon repair (Lu et al., 2017; Yu et al., 2018; Zhao et al., 2022; Chen et al., 2022; Liu et al., 2023). These lncRNAs exert their effects through various mechanisms such as regulating fibroblast proliferation, tenogenic differentiation, and osteogenic differentiation of tendon-derived stem cells. Specifically, XIST promotes fibroblast proliferation and tendon adhesion formation through the miR-26a-5p/COX2 pathway (Chen et al., 2022), while H19 accelerates tenogenic differentiation by targeting miR-29b-3p and activating TGF-β1 signaling (Lu et al., 2017). Additionally, MALAT1, KCNQ1OT1, and AC108925 have been implicated in regulating tenogenic and osteogenic differentiation processes, suggesting their potential as therapeutic targets for tendon injuries. In recent years, there has been increasing evidence that lncRNAs are involved in tendinopathy. Zhang et al. detected 40 differentially expressed lncRNAs in tendinopathy (Zhang et al., 2015); subsequently, Ge et al. constructed a co-expression network that included important genes associated with tendinopathy, including lncRNA NONHSAT209114.1, lncRNA ENST00000577806, lncRNA NONHSAT168 464.1, lncRNA PLK2, lncRNA TMEM214, and lncRNA IGF2 (Ge et al., 2020); Zheng et al. found that eight dysregulated lncRNAs may be involved in fiber formation after tendon injury (Zheng et al., 2018); stable overexpression of lncRNA H19 contributes to TGF-β1-induced acceleration of the tendon differentiation process *in vitro* and promotes rapid tendon healing (Lu et al., 2017). Additionally, it was shown that the LncRNA TUG1 enhances the process of tendon stem/progenitor cell (TSPC) differentiation into bone-forming cells by interacting with the basic fibroblast growth factor (bFGF) protein and facilitating ubiquitination, which ultimately results in the suppression of bFGF protein production (Yu et al., 2020). Currently, studies on lncRNAs are highly favored and researchers combine them with proteins, DNA or RNA to regulate the whole process from pre-transcription to post-transcription. They are also considered to be important pathways for the regulation of mRNA expression and the transcriptional activity of target genes. Previously, Trella et al. found that promoter methylation of lncRNA Mmp25, lncRNA Foxf1, lncRNA Leprel2, lncRNA Igfbp6, and lncRNA Peg12 was differentially methylated in tendinopathies by genome-wide analysis (Trella et al., 2017). It was reported that tendon cell oxidative stress levels were reduced and cell proliferation was enhanced after lncRNA demethylation, suggesting that lncRNA demethylation rescued tendon injury. In addition, they found that adipose-derived MSCs promoted healing after tendon injury by reducing the promoter methylation level of lncRNA Morf4l1 (Zhao et al., 2022). Targeting the lncRNA dnm3os repressed a range of genes associated with fibrogenesis (Zheng et al., 2018). These findings suggest that lncRNAs could be new biomarkers and therapeutic targets.

More and more studies have shown that lncRNAs compete as endogenous RNAs or miRNA sponges, thereby regulating multiple cellular activities. lncRNA MALAT1 has been reported to promote bone cell growth by adjusting the miR-34c/SATB2 axis (Yang et al., 2019). Zhang et al. found that lncRNA MALAT1 is involved in apoptosis, proliferation, and ECM degradation by regulating the miR-150-5p/AKT3 axis (Zhang et al., 2019). Studies have shown that lncRNA MALAT1 acts as a medium for tendon differentiation and repair through the regulation of the miR-378a-3p/MAPK1 axis [89]. Silent lncRNA KCNQ1OT1 regulates the adipogenic and osteogenic differentiation of tendon stem cells through the miR-138/RUNX2 axis, suggesting that it may be a potential therapeutic target for tendon lesions (Zhao et al., 2022). Additionally, lncRNA H19 inhibits tendon differentiation by directly targeting miR-29b-3p and suppressing the expression of TGF-β1 and type I collagen (Lu et al., 2017). Huang et al. constructed a regulatory network of lncRNA-related competing endogenous RNA (ceRNA) and identified seven key lncRNAs associated with tendon disorders. Among these, lncRNA MIR133A1HG may influence the expression of Collagen Type IV Alpha 3 chain (COL4A3) and tropomyosin 3 (TPM3) by competitively binding with hsa-miR-218-1-3p and hsa-miR-1179; downregulated lncRNA C10orf71-AS1 can competitively bind with hsa-miR-130a-5p to regulate the expression of COL4A4, thereby controlling ECM interaction with receptors, focal adhesion, and the PI3K-Akt signaling pathway (Huang et al., 2022). LncRNA AC108925 can regulate the osteogenic differentiation of human tendon-derived stem cells by adjusting miR-146a-3p, suggesting that targeting the lncRNA AC108925/miR-146a-3p axis may be a potential approach for treating tendon disorders. Compared to miRNA, the role of lncRNA in tendon lesions is less studied, and further research is needed to provide new effective targets for the treatment of tendon lesions.

**FIGURE 3:**
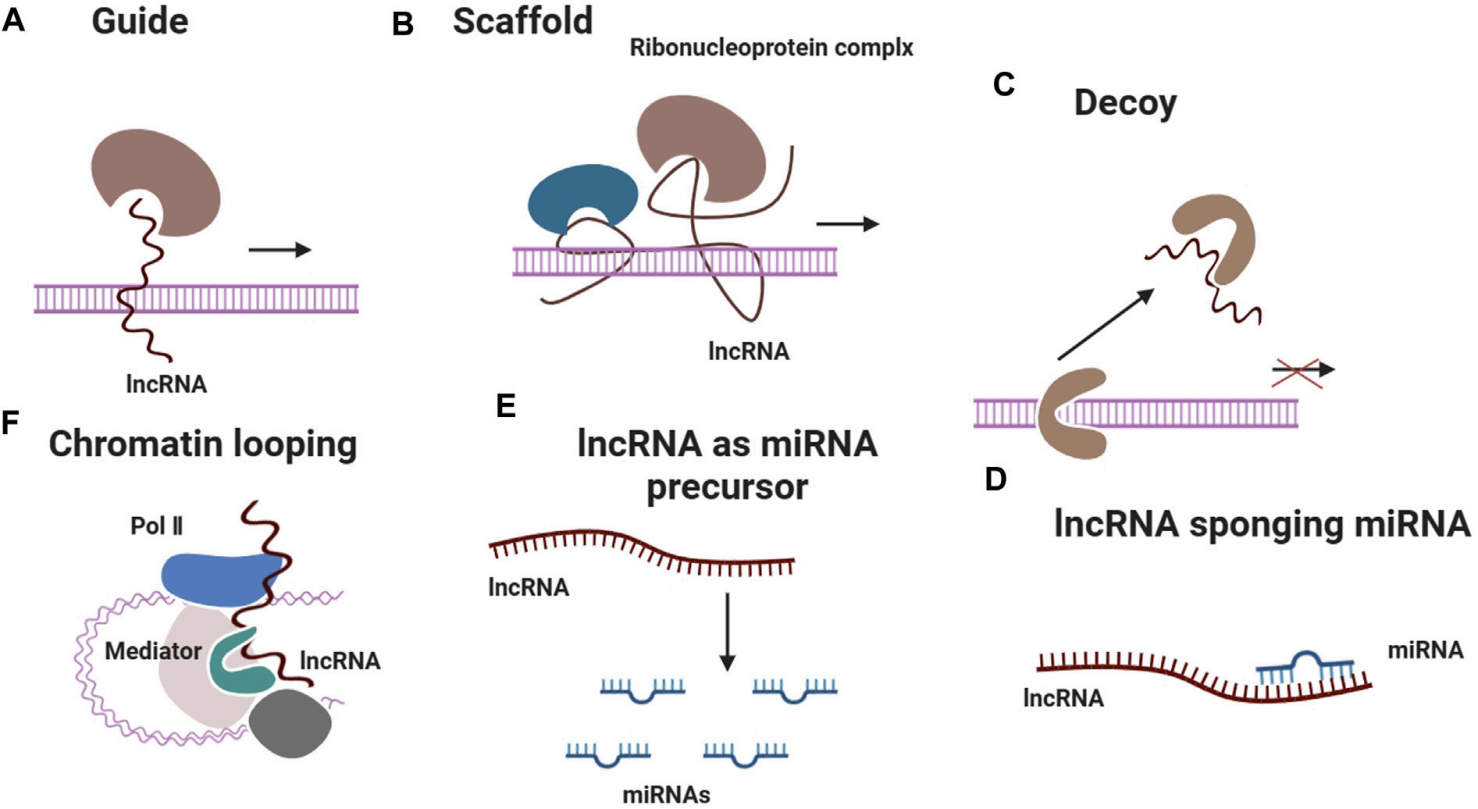
LncRNAs exert regulatory effects on gene expression through diverse mechanisms, including guiding ribonucleoprotein complexes to specific genomic loci, serving as scaffolds for protein complex assembly, acting as decoys to sequester regulatory proteins, sponging miRNAs to modulate their activity, acting as precursors for miRNA biogenesis and facilitating chromatin looping to alter transcriptional programs.

Both miRNAs and lncRNAs are involved in the regulation of gene expression after transcription (Figure 3). miRNAs primarily operate by attaching to the 3′untranslated regions (UTRs) of target mRNAs, which results in their destruction or inhibition of translation (Volovat et al., 2020). On the contrary, lncRNAs have the ability to function through diverse ways, such as acting as scaffolds, decoys, or guides for other molecules. Additionally, they can interact with miRNAs to regulate their function (Volovat et al., 2020). The integration of these two categories of non-coding RNAs has the potential to establish a stronger and more precise regulatory network capable of addressing various facets of the tendon repair process. miRNAs and lncRNAs have been observed to interact with crucial signalling pathways that play a role in cell proliferation, differentiation, and migration. These pathways are vital for tissue healing. An example of this is when the lncRNA Morf4l1 enhances the expression of TGF-β2 by specifically targeting miR-145-5p, which plays a vital role in the growth of tenocytes (Hsu et al., 2023). By integrating miRNAs and lncRNAs that target distinct molecules within the identical pathway, it may be feasible to attain a synergistic impact that amplifies the healing response.

## Summary and outlook

Tendons are complexly composed tissues with unique structures, functions, and mechanics. If a tendon is injured, it will destroy the structure of the tendon, impairing all functions, as well as causing significant pain and even disability. The main complications of healing a tendon injury are adhesions and scarring. Promising results have been achieved in this field, however, there are still a large number of scientific questions that deserve to be explored. Non-coding RNA-based therapies are highly regarded in the field of regenerative medicine because of their many advantages (Sun et al., 2022; Zhang et al., 2022; Zhou et al., 2022). It is promising that the spectrum of noncoding RNAs in tendinopathies and the role of individual noncoding RNAs in tendon repair and healing are being progressively uncovered. Non-coding RNA-based strategies have shown considerable beneficial effects on improving tendon healing in terms of reducing adhesions, promoting remodeling, and facilitating angiogenesis (Zhang et al., 2022; J et al., 2021; Huang et al., 2024; Luo et al., 2022). Although non-coding RNA therapies for tendon repair have shown promise, there are challenges in translating these therapies into clinical application. Key challenges include delivery methods、dosage and timing、safety and efficacy、cost and accessibility and regulatory approval (Zhao et al., 2022). Notwithstanding these obstacles, there have been some encouraging advancements in the industry. Exosomes produced from tendon stem cells have been demonstrated to enhance tendon repair by regulating tenocyte proliferation and migration through miR-144-3p (Wang et al., 2022). In addition, researchers have created nanoparticle-based delivery systems to enhance the dispersion and effectiveness of non-coding RNA treatments (Chen et al., 2020). Although non-coding RNA-based therapies for tendon injuries hold promise, it is essential to carefully consider and address any potential side effects and safety concerns associated with these treatments. The introduction of non-coding RNAs into the body may trigger an immune response (Huang et al., 2023) and off-target effects (Fink and Wolfe, 2011), leading to inflammation or other adverse reactions. Toxicity is another concern, particularly if RNAs are not effectively targeted to the affected area, potentially causing damage to healthy cells and tissues. The long-term effects of these therapies on tendon healing and function are not fully understood. The creation and production of non-coding RNA, as well as its distribution, can be intricate and expensive in terms of economic cost. Optimising manufacturing processes and minimising production expenses will be crucial for enhancing affordability.

The present study on tendon damage repair utilising non-coding RNAs emphasizes the lack of comprehension regarding the control of tendon healing processes and the regulation of pertinent signaling pathways (Lyu et al., 2023). Notable deficiencies include the absence of extensive data on how different cellular and signalling pathways impact the repair of tendons and the control of their constituents (Ke and Zhang, 2023). In order to fill these knowledge gaps, future research should prioritize investigating the interaction patterns and mechanisms between non-coding RNAs and biological scaffolds and their processes in the process of tendon repair. It is reasonable to believe that through the relentless exploration of the above issues, they will be well applied and scarless healing will be achieved.
